# Editorial: Celebrating the International Year of the Periodic Table: Beyond Mendeleev 150

**DOI:** 10.3389/fchem.2020.610869

**Published:** 2020-11-05

**Authors:** Mikhail V. Kurushkin, W. H. Eugen Schwarz, Eugene Goodilin

**Affiliations:** ^1^Chemistry Education Research and Practice Laboratory, SCAMT Institute, ITMO University, Saint Petersburg, Russia; ^2^Chemistry Department, University of Siegen, Siegen, Germany; ^3^Lomonosov Moscow State University, Moscow, Russia

**Keywords:** Iypt 2019, periodic table, Mendeleev 150, periodic system, chemical elements

The Periodic Table of all Chemical Elements has been, for the first time, designed and graphically displayed as a pervasive natural law by the Russian scientist Dmitri Mendeleev in March 1869, followed immediately by a large jump in pure and applied chemistry, physics and other natural sciences, and in education. The Periodic Table of Elements covers most of preceding efforts of other researches on the same subject; it was convincingly supported by the famous quantitative graphs of various elemental properties by Julius Lothar Meyer. During the past one and a half centuries, the Periodic Table has undoubtedly become the true icon of chemistry, having inspired numerous scientists of various specialities, philosophical thinkers, bel-esprits and bread-and-butter scholars as well. Thereby it has opened up new exciting horizons for innovative sustainable developments and improved advances of humankind. Periodic Tables have become an integral part of basic natural sciences, of all chemical classrooms, and even of popular culture and arts across the globe (Ball, 2019; Chapman, 2019; Düllmann, 2019; Goodilin et al., 2019; Gorbunova et al., 2019; Liddle, 2019; Poliakoff et al., 2019; Radford, 2019; Rahm et al., 2019; Rampling, 2019; Scerri, 2019; Shaik et al., 2019).

On this background, the United Nations General Assembly proclaimed the International Year of the Periodic Table of Chemical Elements (IYPT 2019) to commemorate the 150th anniversary of the discovery of the Natural System of Chemical Elements as one of the most important and influential achievements in modern sciences. This provided an unparalleled opportunity to highlight the continuous nature of scientific discovery in a peaceful and enlightened world. Advances in research in many different contexts were triggered by the discovery of the Natural System, yielding good ground for recognizing the importance of chemistry in cooperation with other basic disciplines in a world endangered by climate change and unlimited population increase.

The present collection of Research Topics is dedicated to the Natural System of Elements and its various representations in the form of Periodic Tables, its scientific chemical and physical foundations, its innovative applications in laboratory research, its cutting-edge findings in chemical periodicity and elemental groups, in advanced chemical research, physics, chemical engineering, material science, catalysis, environmental science, photovoltaics, and memristors.

Cao et al. discuss the chemical elements in their review as the “kernel” that is conserved when substances are altered. Graphical displays of the chemical properties of the elements, in the form of Periodic Tables, have been designed with the aim of either classifying real chemical substances or emphasizing formal and aesthetic concepts. The former tables usually incorporate typical valence electron configurations of bonded atoms in chemical compounds, instead of the common but chemically atypical ground states of free atoms in physical vacuum; basic chemical properties like valence numbers, size and energy of the valence shells with their joint variation over the elements showing principal and secondary periodicity; peculiar elements at the top and at the bottom of the Periodic Table. While it is essential that Periodic Tables display important trends in element chemistry we might expect unusual chemical behavior in ambient, near ambient, or unusual conditions. The combination of experimental data and theoretical insight supports a more nuanced understanding of the complex periodic trends and the non-periodic phenomena.

As an example of the rich chemistry of elements and its pronounced influence on fundamental properties and structural correlations for modern materials, Belich et al. compare several revolutionary materials based on the perovskite type of crystal lattices including the legendary families of high-temperature superconductive cuprates, of colossal magnetoresistive manganites and of hybrid lead halides for a new generation of solar cells. The authors demonstrate that the widely spread crystal lattices of perovskites represent a natural flexible platform for chemical design of various advanced functional materials with unique features. An interplay between chemical bonding, defects and crystal chemistry peculiarities makes the perovskite structure a “lego designer” utilizing the natural features of the chemical elements.

Several authors presented their ideas advancing important classes of materials and nanomaterials from the C, Si, Ti, Sn, Pb family of group IV elements of the classical form of the Periodic Table. Navrotskaya et al. describes deeply modified 1D carbon nanomaterials, focusing on hybrid nanomaterials with unique mechanical, electrical, thermal, and optical characteristics. Among them, hybrids based on filamentous forms of carbon, such as carbon nanotubes and carbon nanofibers, in combination with inorganic nanoparticles attract particular attention. Due to the structure and morphology, charge, and energy transfer processes lead to synergistic effects that allow the use of less material with higher productivity including their ecological applications.

Morozova et al. discuss an important class of silicon nanomaterials—quantum dots. The authors noted that widely known silicon quantum dots (SiQDs), semiconductor Si nanoparticles ranging from 1 to 10 nm, still hold a great applicative potential for optoelectronic devices and fluorescent bio-marking agents. Their much higher biocompatibility, as compared to conventional toxic Group II-VI and III-V metal-based quantum dots, makes their practical applications even more attractive to prevent environmental pollution and to avoid damage of living organisms.

Oxide derivatives of silicon composes a special class of glass materials. In particular, Shakhgildyan et al. shared a perspective of how glass is the only material that could represent almost all elements of the Periodic Table inside itself, showing the effect of the Periodic Law on properties of the final material. The authors reproduced for the first time the Periodic Table in birefringence colors in the bulk of silica glass using a direct laser writing technique.

Highly important and technologically relevant oligosiloxanes are discussed by Rabanzo-Castillo et al.. In particular, the utility of (C_6_F_5_)_3_B(OH_2_) as a catalyst for the simple and environmentally benign synthesis of oligosiloxanes directly from hydrosilanes, is discussed. The authors note that this protocol offers several advantages compared to other methods of synthesizing siloxanes, such as mild reaction conditions, low catalyst loading, and a short reaction time with high yields and purity, although such reactions have a rather complicated mechanism. With the hydrosilane (R_3_SiH) as the sole starting material, the fate of the reaction largely depends on the creation of silanol (R_3_SiOH) from R_3_SiH as these two undergo dehydrocoupling to yield a disiloxane product. Generation of the silanol is based on a modified Piers-Rubinsztajn reaction. Once the silanol has been produced, the mechanism involves a series of competitive reactions with multiple catalytically relevant species involving water, silane, and silanol interacting with the Lewis acid, and the favored reaction cycle depends on the concentration of various species in solution.

Rodionov et al. describe improved access to expected applications of titania and its derivatives for photocatalytic systems. The authors present new preparation routes, final structural and functional properties of layered hybrid derivatives of titania as an efficient photocatalyst for hydrogen production from aqueous alcohol solution. The hybrid photocatalyst H_2_Nd_2_Ti_3_O_10_ × BuNH_2_ was synthesized by a solid-state ceramic method followed by protonation, intercalation of methylamine and subsequent substitution by n-butylamine. While the non-intercalated Pt-loaded H_2_Nd_2_Ti_3_O_10_ showed a maximum quantum efficiency of only 2% in the 220–340 nm range, the efficiency for hybrid samples reached 23–52%. This effect may be associated with the significant expansion of the interlayer space, which is considered as a separate reaction zone.

In addition, Voytovich et al. compare titanates and their role as photocatalytic splitting systems with a similar series of hybrid niobates HCa_2_Nb_3_O_10_ × RNH_2_, containing intercalated n-alkylamines (R = Me, Et, Pr, Bu, Hx, Oc). Special attention was also paid to the feasible improvement of the photocatalytic activity of the samples via their modification with Pt nanoparticles as a cocatalyst. Thus, modification of layered perovskite-like oxides by organic substances appears to be an effective strategy to manage their photocatalytic activity, which may be applied to other related photocatalytic materials.

Illarionov et al. review state-of-the-art memristive titania as one of the most widely used materials in resistive switching applications, including random-access memory, neuromorphic computing, biohybrid interfaces, and sensors. The functional memristive properties of titanium dioxide thin films are drastically dependent on their processing methods, including synthesis, fabrication, and post-fabrication treatment, and thus the authors provide an overview of the major application domains of TiO_2_-based memristive devices.

In their mini-review, Petrov and Tarasov summarize the existing data on the unique properties of reactionary polyiodide melts (RPM) used for the development of the novel type of technologies of scalable production of a new generation of solar cells based on hybrid halloplumbate perovskites. The authors discuss the potential of RPM for the fabrication of hybrid perovskite films and describe the role of polyhalides the degradation of perovskite solar cells.

Another potential candidate for photovoltaics based of halostannates is presented in the article of Umedov et al.. Structure and properties of inorganic perovskite Cs_2_SnI_6_ demonstrate its potential as a light-harvester or electron-hole transport material. The authors report the way of light tuning of absorption and transport properties of cesium iodostannate(IV) Cs_2_SnI_6_ via partial heterovalent substitution of tin for indium. Light absorption and optical bandgaps of such materials have been investigated by UV-VIS absorption and photoluminescent spectroscopies. Low-temperature electron paramagnetic resonance spectroscopy was used to study the kind of the paramagnetic centers in these materials.

A further domain of articles in this Collection is closely connected with f-elements. A quite original mini-review of radiochemistry experts Romanchuk et al. disclose some most important mainstream problems in radio-ecology. The review briefly but comprehensively analyzes the highly complicated chemistry of the actinides and their applications and utilization. Among them, uranium and plutonium are the most important, as they are used in the nuclear fuel cycle and nuclear weapon production, and thus the review is focused on the latest experimental, modeling and case study achievements in the investigation of plutonium and uranium migration in the environment, which include the speciation of these elements and the chemical reactions that control their migration pathways.

Visentin et al. presented the current status of gaseous transport studies of singly-charged lanthanide and actinide ions in the light of potential applications to superheavy ions. The correlation of the ion-neutral interaction potential and mobility variations with the spatial parameters of the electron distributions in the bare ions is explored through the ionic radii concept. While the qualitative trends found for interaction potentials and mobilities render them appealing for superheavy ion research, lack of experimental data and limitations of the scalar relativistic ab initio approaches in use make further efforts necessary to bring the transport measurements into the inventory of techniques operating in the “one atom at a time” mode.

Finally, Elkina and Kurushkin authored a comprehensive mini-review on the history, the different syntheses, and possibly all known applications of artificial element promethium. The mini-review is expected to be the definitive starting point for anyone interested in learning about the only chemical element in the lanthanide series that has no stable isotopes.

Overall, the Collection summarizes various new achievements and trends in the chemistry of elements over the Periodic System and is certainly useful for chemists and practical researchers in materials science as well as sustainable chemistry.

## Author Contributions

All authors listed have made a substantial, direct and intellectual contribution to the work, and approved it for publication.

## Conflict of Interest

The authors declare that the research was conducted in the absence of any commercial or financial relationships that could be construed as a potential conflict of interest.
